# SPC24 promotes osteosarcoma progression by increasing EGFR/MAPK signaling

**DOI:** 10.18632/oncotarget.22167

**Published:** 2017-10-27

**Authors:** Jun Sheng, Mengchen Yin, Zhengwang Sun, Xia Kang, Da Liu, Kai Jiang, Jia Xu, Feixing Zhao, Qunfeng Guo, Wei Zheng

**Affiliations:** ^1^ Department of Orthopedics, Chengdu Military General Hospital, Chengdu, Sichuan, China; ^2^ Department of Orthopedics, Longhua Hospital, Shanghai University of Traditional Chinese Medicine, Shanghai, China; ^3^ Department of Orthopedics, Changzheng Hospital, The Second Military Medical University, Shanghai, China; ^4^ Department of Personnel Office, Traditional Chinese Medical Hospital of Zhuji, Zhuji, Zhejiang, China; ^5^ Department of Pathology, Zhuji People's Hospital of Zhejiang Province, Zhuji, Zhejiang, China

**Keywords:** SPC24, osteosarcoma, Ras/Raf/MEK/ERK signal pathway, E-cadherin

## Abstract

In this study, we investigated the role of the spindle checkpoint protein SPC24 in osteosarcoma progression. SPC24 knockdown in 143B and U2OS osteosarcoma cells decreased cell growth, survival and invasiveness. The SPC24 knockdown cells also exhibited low EGFR, Ras and phospho-ERK levels and high E-cadherin levels, suggesting inhibition of EGFR/Ras/ERK signaling and epithelial-to-mesenchymal transitioning. Xenografted SPC24 knockdown osteosarcoma cells showed reduced tumor growth in nude mice with decreased EGFR and phospho-ERK levels and increased E-cadherin levels. By contrast, human osteosarcoma tissue samples showed high SPC24 and phospho-ERK levels and low E-cadherin levels. These results suggest SPC24 promotes osteosarcoma progression by increasing EGFR/Ras/ERK signaling.

## INTRODUCTION

Osteosarcoma (OS) is a malignant solid bone tumor that is highly malignant with low survival rates [1, 2]. Nearly 30-40% of the patients with localized osteosarcoma undergo relapse due to lung metastasis [3]. The survival rates of patients with metastatic OS is below 30%[4, 5]. The 5-year survival rate of OS patients is 55-70% despite improved surgical procedures and multidrug chemotherapy [6, 7]. Therefore, there is an urgent need to identify new therapeutic and prognostic biomarkers associated with osteosarcoma progression and metastasis.

Many cancers show mutations of receptor tyrosine kinesis (RTKs) resulting in aberrant Ras/ERK signaling [8]. The Ras/ERK signaling pathway regulates cellular survival, growth and differentiation [9]. Nearly 67% osteosarcomas show aberrant ERK activation [10]. Osteosarcomas also show upregulated RTKs such as EGFR [11]. Targeted inhibition of overexpressed or mutant RTKs has diminished therapeutic outcomes due to compensatory activation of other RTKs [12]. MEK inhibitors demonstrate therapeutic potential in human osteosarcoma cells due to constitutive ERK activation [3].

Nuclear division cycle 80 (Ndc80) is a kinetochore complex made up of two heterodimers, CDCA1-KNTC2 and SPC24-SPC25 [13]. It is essential for stable kinetochore-microtubule anchoring and normal chromosomal segregation during mitosis [14]. During mitosis, the SPC24/SPC25 protein complex anchors the Ndc80 complex to the inner kinetochore with the nuclear spindle microtubules and kinetochores [13, 15]. Disruption of spindle checkpoint proteins alters chromosomal segregation resulting in genetic instability and aneuploidy [16]. The spindle checkpoint proteins, SPC24 and SPC25 are implicated in colorectal and hepatocellular carcinomas [14, 17]. In this study, we investigated the role of SPC24 in osteosarcomagenesis.

## RESULTS

### SPC24 knockdown decreases growth, proliferation and colony formation

**Figure 1:**
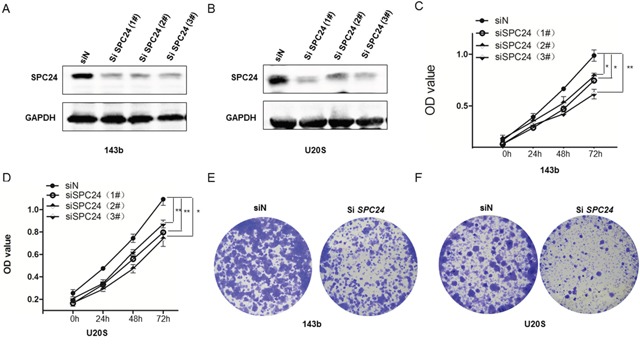
SPC24 knockdown decreases growth, viability and colony formation in 143B and U2OS cells **(A-B)** Representative western blot images showing SPC24 expression in siSPC24 and siN transfected (A) 143B and (B) U2OS cells. β-actin is the loading control. **(C-D)** MTT assay showing viability of siSPC24 and siN transfected (C) 143B and (D) U2OS cells at 24, 48 and 72 h. **(E-F)** Soft-agar colony formation assay showing crystal violet stained colonies from siSPC24 and siN transfected (E) 143B and (F) U2OS cells. Note: ^*^ denotes p < 0.05 compared to control; ^**^denotes p < 0.01 compared to control.

Western blot analysis revealed that U2OS and 143B cell lines transfected with three different SPC24 siRNAs showed lower SPC24 protein levels than controls (Figure 1A-1B). MTT assay demonstrated that SPC24-knockdown (siSPC24) U2OS and 143b cells showed decreased cell proliferation than control cells at 24, 48, and 72 h (Figure 1C-1D). Moreover, SPC24 knockdown U2OS and 143b cells showed decreased colony formation (Figure 1E-1F, Supplementary Figure 1A).

### SPC24 knockdown promotes apoptosis and cell cycle arrest in OS cells

**Figure 2:**
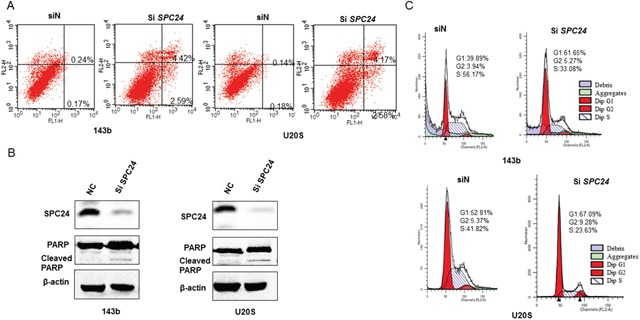
SPC24 knockdown promotes apoptosis and G1-S cell cycle arrest in 143B and U2OS cells **(A)** Flow cytometry analysis of AnnexinV-FITC and propidium iodide stained control (siN) and SPC24 knockdown 143B and U2OS cells. AnnexinV^+^ PI^−^ cells and AnnexinV^+^ PI^+^ cells represent early and late apoptotic cells, respectively. **(B)** Representative western blot showing SPC24 and PARP/cleaved PARP levels in control and siSPC24 transfected OS cells at 72 h. **(C)** Flow cytometry analysis of cell cycle distribution in control (siN) and SPC24 knockdown 143B and U2OS cells. Cells were stained with propidium iodide.

Dysregulated apoptosis is the hallmark of cancer cells. We evaluated the effects of SPC24 knockdown on OS cell apoptosis using AnnexinV/propidium iodide double staining. Flow cytometry analysis demonstrated that SPC24 knockdown increased early (AnnexinV^+^ PI^−^) and late (AnnexinV^+^ PI^+^) stage apoptosis (Figure 2A, Supplementary Figure 1B).

Moreover, SPC24 knockdown cells showed higher cleaved PARP levels than in control cells, further demonstrating increased apoptosis (Figure 2B).

Disrupted SPC24 results in uncontrolled mitosis because the cells are spindle checkpoint defective [18]. Therefore, we analyzed the cell cycle of SPC24 knockdown OS cells. Flow cytometry analysis showed that SPC24 knockdown demonstrated increased G1 phase cells and decreased S and G2-M phase cells than in control (siN) OS cells suggesting G1-S cell cycle arrest (Figure 2C and Supplementary Figure 1C).

### SPC24 activated EGFR/Ras/Raf/MEK/ERK signal pathway

**Figure 3:**
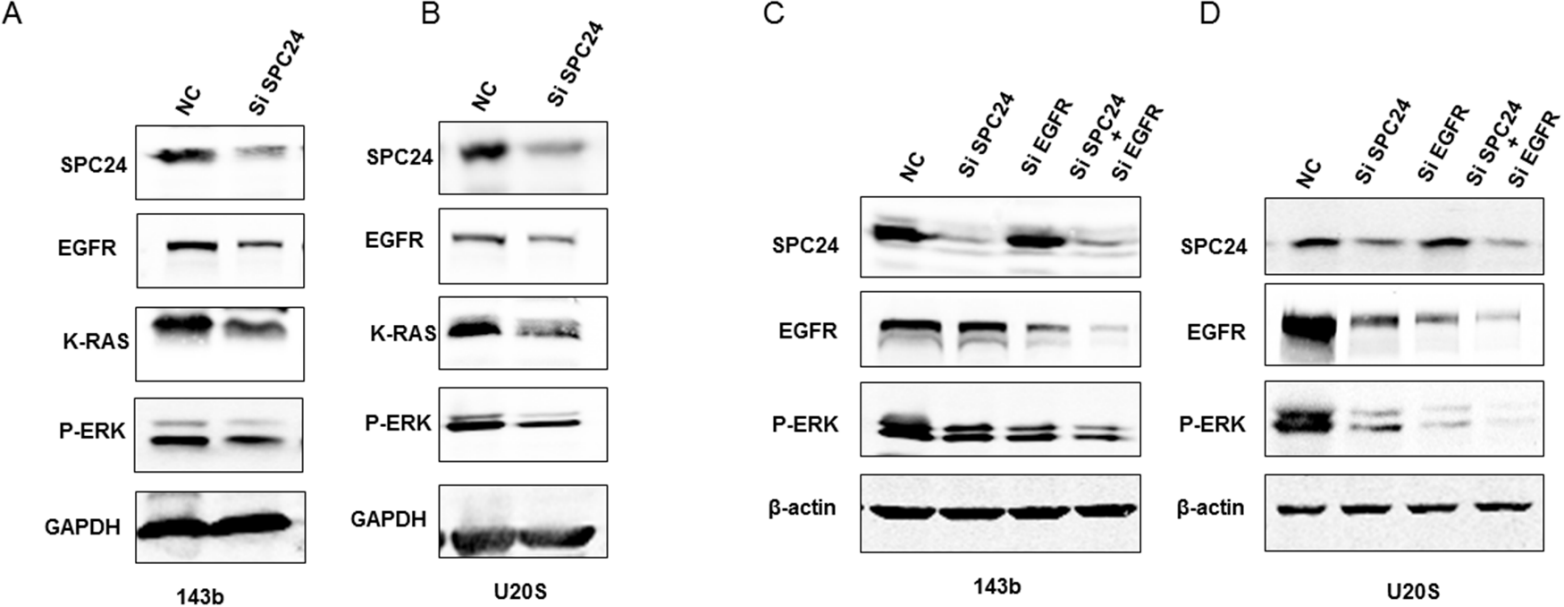
SPC24 knockdown inhibits EGFR/Ras/ERK signaling in 143B and U2OS cells **(A-B)** Representative western blot showing SPC24, EGFR, K-Ras and p-ERK levels in siN or siSPC24 transfected (A) 143B and (B) U2OS cells. **(C-D)** Representative western blot showing SPC24, EGFR, K-Ras and p-ERK levels in siN or siEGFR transfected (A) 143B and (B) U2OS cells.

Pancreatic, colon, lung, ovarian and kidney tumors show aberrant epidermal growth factor receptor (EGFR) activation and MAP kinase signaling [19, 20]. We determined the effect of SPC24 knockdown on the EGFR/Ras/ERK signaling pathway. SPC24 knockdown decreased EGFR, Ras and p-ERK expression in OS cells than in controls (Figure 3A-3B). Howeve, the decreased level of p-ERK was not obvious with EGFR knockingdown when SPC24 was silenced (Figure 3C-3D).

### SPC24 knockdown decreases OS cell invasiveness and EMT

**Figure 4:**
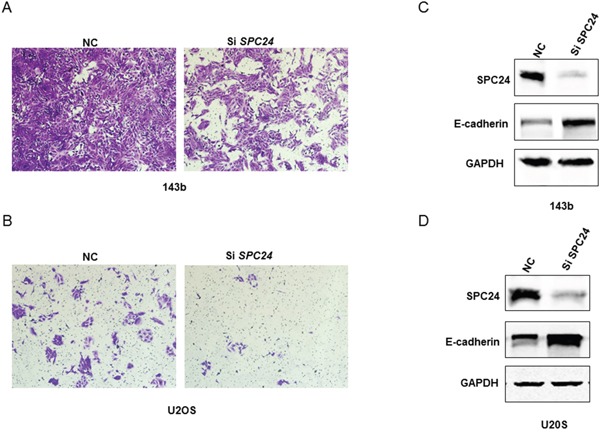
SPC24 knockdown increases invasiveness and EMT in 143B and U2OS cells **(A-B)** Representative images (250X) showing transwell invasion assay results of siN or siSPC24 transfected 143B and U2OS cells. **(C-D)** Representative western blots showing E-cadherin and SPC24 levels in siN or siSPC24 transfected 143B and U2OS cells at 72 h.

Next, we investigated the role of SPC24 in osteosarcoma metastasis by Transwell invasion assays. SPC24 knockdown in U2OS and 143b cells decreased cell migration (Figures 4A-4B and Supplementary Figure 1D).

E-cadherin plays a critical role in epithelial to mesenchymal transitioning (EMT), which is a key step in cancer metastasis and invasiveness. Therefore, we determined the effect of SPC24 knockdown on E-cadherin levels. SPC24 knockdown OS cells showed high E-cadherin expression than control cells (Figure 4C-4D). This suggested that SPC24 mediated osteosarcoma metastasis by downregulating E-cadherin.

### SPC24 knockdown inhibits xenograft OS tumor growth in nude mice model

**Figure 5:**
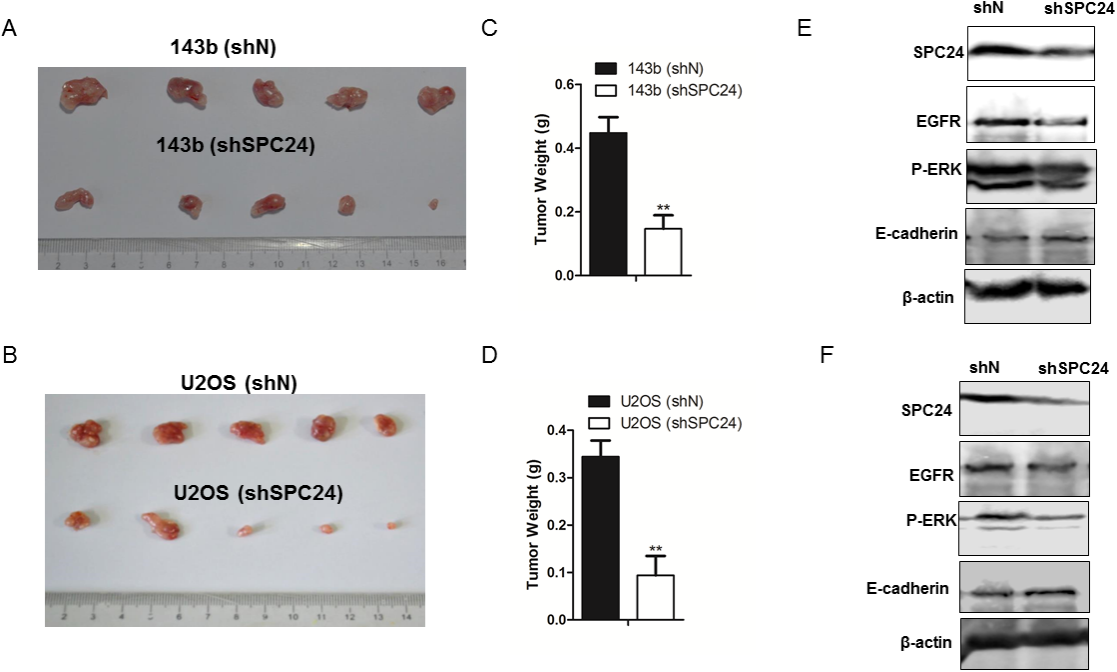
SPC24 knockdown inhibits osteosarcoma xenograft tumor growth in nude mice model **(A-B)** Representative western blot analysis showing SPC24 expression in shN and shSPC24 transfected 143b and U2OS cells subcutaneously injected into 5-week old nude mice. (B) The tumor weights of xenograft tumors derived from control and SPC24 knockdown 143B and U2OS cells. Note: Values represent mean ± SEM; ^**^ denotes p< 0.01 compared to control. **(C-D)** Representative western blot showing SPC24, EGFR, p-ERK and E-cadherin levels in xenograft tumors derived from control and SPC24 knockdown 143B and U2OS cells.

Next, we investigated the effects of SPC24 knockdown on *in vivo* osteosarcoma progression by xenografting control and SPC24 knockdown OS cells into dorsal flanking sites of 5 week old nude mice. We observed that the size and weight of shSPC24 tumors were highly reduced than shN tumors (p<0.01; Figure 5A-5D). Western bolt analysis demonstrated decreased EGFR and p-ERK as well as increased E-cadherin in shSPC24 tumors than shN tumors (Figure 5E-5F).

### OS patient tissues show high SPC24 protein levels

**Figure 6:**
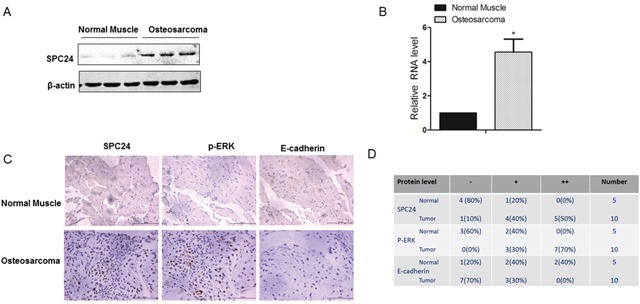
Human osteosarcoma patient tissues show increased SPC24 and p-ERK and decreased E-cadherin levels **(A)** Representative western blot shows SPC24 levels in normal and OS patient tissue samples. B-actin was used as control. **(B)** QRT-PCR analysis of SPC24 mRNA levels in normal and OS patient tissue samples. **(C)** Representative images (magnification: 20x) show immunohistochemical staining of SPC24, p-ERK and E-cadherin in normal and OS tissue samples (Scale bar, 125μm). **(D)** Semiquantitative analysis of SPC24, p-ERK and E-cadherin expression in normal and OS cancer tissue samples based on immunohistochemical staining.

Next, we analyzed the correlation between SPC24 and EGFR/Ras/ERK signaling pathway in human OS samples. OS patient tissue samples showed high SPC24 mRNA and protein expression than in normal tissues (Figure 6A-6B). Moreover, OS patient tissues showed increased SPC24 and p-ERK, which correlated with decreased E-cadherin based on IHC staining (Figure 6C-6D).

## DISCUSSION

Osteosarcoma (OS) is the most common bone tumor. Osteosarcoma patients with pulmonary metastasis have a survival rate of <20% despite aggressive surgery and intensive chemotherapy [21]. Hence, identifying critical pathways regulating osteosarcoma metastasis is of paramount importance to develop novel therapeutic strategies. Human osteosarcoma xenograft models have been used to identify genetic factors and proteins that are associated with tumor progression and metastasis [22]. In this study, we demonstrate the oncogenic role of SPC24 in osteosarcomagenesis.

Disruption of spindle checkpoint proteins disrupts chromosomal segregation resulting in genetic instability and aneuploidy [16]. The changes in chromosomal copy number (aneuploidy) and aberrant chromosomal segregation contribute directly to the development and metastasis of malignant tumors [14, 23, 24]. SPC24 is a core component of the Ndc80 kinetochore complex, which is essential for directional movement of the chromosomes to the spindle poles during anaphase [25]. We demonstrate that SPC24 knockdown OS cell lines show decreased cell growth and proliferation and increased apoptosis.

Yu *et al*. reported that aberrant hyperactivation of the Ras/Raf/MEK/ERK pathway promoted lung metastasis in mice osteosarcoma model. Our study demonstrated that SPC24 activated EGFR/Ras/ERK signal pathway in osteosarcoma cells. The Ras/Raf/MEK/ERK pathway is upregulated in nearly 30% of human cancers [26, 27] and regulates metastasis in various cancers [28]. EMT is a key process that regulates cancer metastasis [29]. SPC knockdown in OS cells results in higher E-cadherin expression. These data demonstrate that SPC24 promotes invasiveness in OS by downregulating E-cadherin.

We also demonstrated that SPC24 knockdown OS cells reduced xenograft tumor growth. Moreover, they showed reduced p-ERK and higher E-cadherin levels. Furthermore, human OS samples showed high SPC24 and p-ERK expression as well as low E-cadherin. In conclusion, we demonstrate that SPC24 is a potential therapeutic target because it regulates OS progression and metastasis by activating EGFR/Ras/ERK signaling and downregulating E-cadherin.

## MATERIALS AND METHODS

### Cell lines

U2OS cells (ATCC) were grown in DMEM (Gibco) with 10% fetal bovine serum (FBS). 143B cells (Beijing Jishuitan Orthopaedic laboratory) were grown in RPMI 1640 (Hyclone) with 10% FBS.

### *SPC24* knockdown in U2OS and 143B cell lines

The stable SPC24 knockout U20S and 143B cell lines were generated by transfecting retroviral shRNA vectors specific for SPC24 (OriGene, Rockville, MD).

For transient SPC24 knockdown, 3 siRNAs against SPC24, (1) 5′-GAGCCUUCUCAAUGCGAAGTT-3; (2) 5′-CCGAGAAGCAGCUGCGAGATT-3′; and (3) 5′-UACCACCAA GUUAGUAAAATT-3′ [14] were transfected with TransExcellent (Cenji Biotech, Shanghai, China) according to manufacturer's instructions. SPC24 mRNA and protein expression in the transfected cells was analyzed by qRT-PCR and western blot at 72 h.

### Transwell invasion assay

Transwell matrigel assay was used to determine OS cell invasiveness as described previously [30]. In the upper part of the transwell chamber, 5×10^4^ cells were seeded in DMEM or RPMI medium without FBS, whereas DMEM or RPMI medium with 10% FBS was added in the lower transwell chamber. After 24 h, the cells on the upper side of the transwell membrane were removed by cotton swabs. The migrating cells on the lower side of the insert filter were fixed with 10% buffered formaldehyde, stained with 1% crystal violet and quantified with a light microscope (Olympus).

### MTT assay

OS cells (1 × 10^4^ cells per 0.5 ml per well) were grown in 24-well plates at 37°C and 5% CO2. The cells were transiently transfected with control or SPC24 siRNAs for 24, 48, and72 h. At each time point, the cells were incubated with 500μg 3-(4,5-dimethyl-thiazol-2-yl)-2,5-diphenyltetrazolium bromide (MTT). Then, the MTT-formazan crystals were dissolved in 500 μl DMSO and the absorbance was measured at 570 nm in an ELISA reader.

### Apoptosis assay

Cell apoptosis was determined by AnnexinV-FITC/PI Apoptosis Detection Kit (Abcam, Cambridge, MA, USA) according to the manufacturer's protocols. The cells were washed in ice-cold PBS, re-suspended in 200 μl binding buffer and incubated with 5 μl Annexin V-FITC and 5 μl propidium iodide (PI) for 15 min at 4°C in the dark. Then, the cells were analyzed by flow cytometry.

### Xenograft animal model

SPC24-knockdown and control cells (2×10^6^ cells/100 μl) were injected into the dorsal flanking sites of 5 week old female BALB/c nude mice. After 3 weeks, the mice were euthanized and the tumors were harvested and used for both biochemical as well as immunohistochemical (IHC) staining. IHC staining was performed on paraffin embedded samples that were sectioned and stained as described.

### Immunohistochemistry (IHC)

Serial paraffin embedded sections from tumor and normal tissues were deparaffinized in xylene and rehydrated in a graded ethanol series. Then, the sections were incubated with antibodies against SPC24, p-ERK and E-Cadherin overnight at 4°C. After PBS washes, the sections were incubated with HRP-conjugated secondary antibody for 1 h at room temperature. Then, the sections were developed with 3, 3-diaminobenzidine tetrahydrochloride (DAB) and counterstained with hematoxylin.

### Statistical analysis

Data are presented as mean ± s.e.m. from at least three independent experiments. Difference between 2 groups was determined by student's t-test. Difference at different time points among groups was evaluated by ANOVA. P < 0.05 was considered statistically significant.

## SUPPLEMENTARY MATERIALS FIGURE


