# Supplementary material for: SPC24 promotes osteosarcoma progression by increasing EGFR/MAPK signaling
Source: Oncotarget. 2017 Oct 27;8(62):105276–83. doi: 10.18632/oncotarget.22167 (PMC5739637; doi:10.18632/oncotarget.22167)
Supplement: Supplementary file 1 [file oncotarget-08-105276-s001.pdf]

SPC24 promotes osteosarcoma progression by increasing EGFR/ MAPK signaling

SUPPLEMENTARY MATERIALS

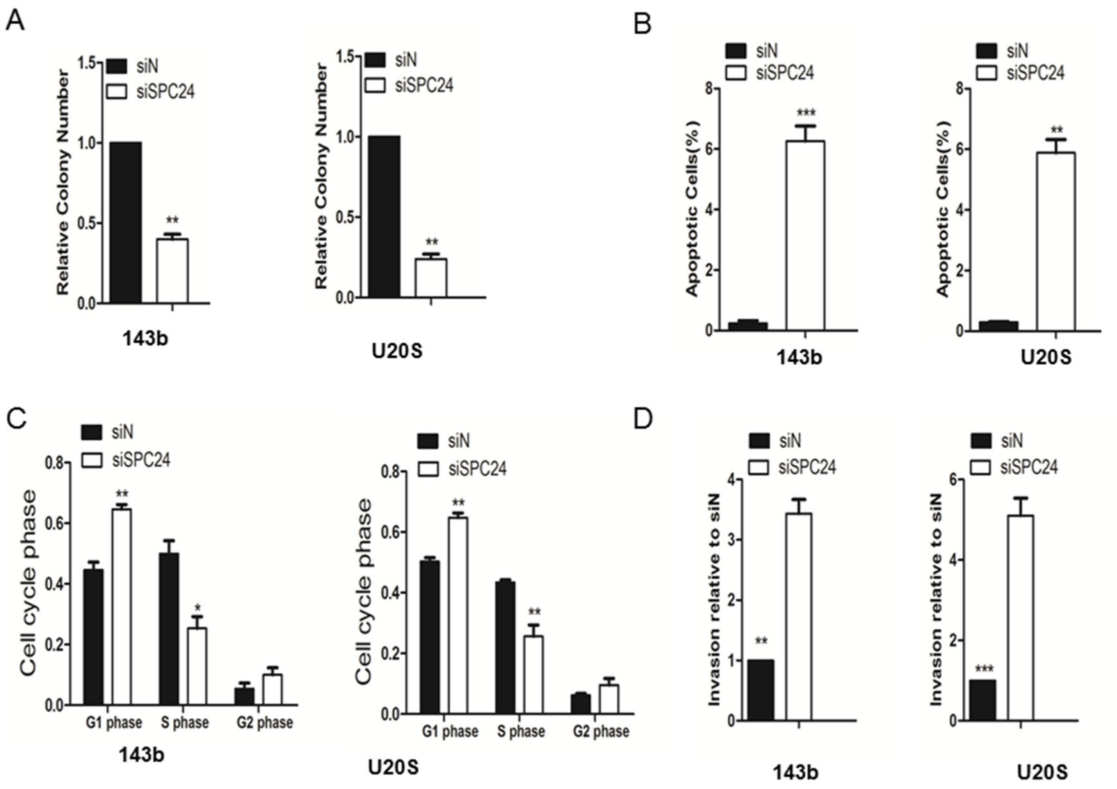

Supplementary Figure 1: (A) The statistical analysis of cell colony assays. Data expressed as the percentage of cell colony represented mean ± SEM of three experiments. $^{**}P < 0.01$. **(B)** The statistical analysis of cell apoptosis assays. Data expressed as the percentage of cell colony represented mean ± SEM of three experiments. $^{**}P < 0.01$; $^{***}P < 0.001$. **(C)** Quantitated results of cell cycle analysis were performed in OS cells with flow cytometry. $^{*}P < 0.05$; $^{**}P < 0.01$. **(D)** The statistical analysis of cell invation. $^{**}P < 0.01$; $^{***}P < 0.001$.
